# In Vitro Degradation of Absorbable Zinc Alloys in Artificial Urine

**DOI:** 10.3390/ma12020295

**Published:** 2019-01-18

**Authors:** Sébastien Champagne, Ehsan Mostaed, Fariba Safizadeh, Edward Ghali, Maurizio Vedani, Hendra Hermawan

**Affiliations:** 1 Department of Mining, Metallurgical and Materials Engineering, Laval University, 1065 avenue de la Médecine, Québec, QC G1V 0A6, Canada; sebastien.champagne.2@ulaval.ca (S.C.); fariba.safizadeh.1@ulaval.ca (F.S.); edward.ghali@gmn.ulaval.ca (E.G.); 2 Research Center of CHU de Québec, 10 rue de l’Espinay, Québec, QC G1L 3L5, Canada; 3 Department of Mechanical Engineering, Politecnico di Milano, Via La Masa 1, 20156 Milan, Italy; ehsan.mostaed@polimi.it (E.M.); maurizio.vedani@polimi.it (M.V.)

**Keywords:** absorbable, corrosion, degradation, magnesium, ureteral stent, zinc

## Abstract

Absorbable metals have potential for making in-demand rigid temporary stents for the treatment of urinary tract obstruction, where polymers have reached their limits. In this work, in vitro degradation behavior of absorbable zinc alloys in artificial urine was studied using electrochemical methods and advanced surface characterization techniques with a comparison to a magnesium alloy. The results showed that pure zinc and its alloys (Zn–0.5Mg, Zn–1Mg, Zn–0.5Al) exhibited slower corrosion than pure magnesium and an Mg–2Zn–1Mn alloy. The corrosion layer was composed mostly of hydroxide, carbonate, and phosphate, without calcium content for the zinc group. Among all tested metals, the Zn–0.5Al alloy exhibited a uniform corrosion layer with low affinity with the ions in artificial urine.

## 1. Introduction

In 2008, up to five in 10,000 Canadian adults suffered from acute urinary tract obstruction, a kidney-related disease contributing to the economy burden of Canada by $3.8 billion [1,2]. It is a blockage that limits the flow of urine out of the body, which can be caused by a variety of factors such as a kidney stone, a swollen prostate, or a tumor [3]. To quickly relieve the kidney of the built-up of urine, urologists will mechanically open the urinary tract using a stent. An ideal ureteral stent will maintain an excellent urine flow to optimize upper tract drainage, be resistant to infection and encrustation (calcification), and be absorbable [4]. The majority of urologists consider the placement of stents after ureteral dilation to be routine. Some studies showed that about 60–85% of urologists maintained the stent for less than seven days [5,6]. This temporary need has brought the concept of biodegradable ureteral stent. Introduced in the late 1980s, their purpose is especially for temporary treatment such as for patients waiting for the effect of a medical therapy [7]. Poly(glycolic acid), poly(lactic acid), and poly(lactic-*co*-glycolic acid) are among the biodegradable polymeric materials used for ureteral stents [4,8]. However, polymeric stents possess a limited ability to resist external compression forces such as those created in a malignant extrinsic ureteral obstruction. This is due to their intrinsic low strength, which often results in their reinforcement by a metal skeleton made of non-degradable alloys [9,10]. At this point, metals that can corrode would constitute the ideal materials for biodegradable ureteral stents.

Nowadays, with the recent development in new biomaterials technology, namely absorbable metals, this ideal ureteral stent should be feasible. Absorbable metals, also known as biodegradable metals, are metals that corrode gradually in vivo with an appropriate host response, then dissolve completely while assisting tissue healing [11]. Iron (Fe), magnesium (Mg), zinc (Zn), and their alloys are among the studied absorbable metals, mostly for cardiovascular and orthopaedic applications. Coronary stents made of Fe and its alloys have shown their safety and efficacy when tested in animals [12], while those made of Mg alloys were clinically tested in humans, showing a continuous favorable safety profile up to 12 months [13]. Zn and its alloys have been more recently proposed as alternatives to Fe and Mg. In recent in vivo studies, pure Zn wire showed a steady corrosion rate for up to 20 months post-implantation in murine artery without causing local toxicity [14], while pure zinc stents corroded in rabbit abdominal aorta without obvious accumulation of corrosion products even after 12 months of implantation [15].

Application of absorbable metals in urology is a novel avenue. In 2014, Lock et al. [16] conducted a study on the degradation and antibacterial properties in artificial urine of Mg, Mg–Y, and Mg–3Al–1Zn (AZ31) that was the first to show the potential of absorbable metals for urological applications. More recent work by Zhang et al. [17,18] reported that implantation of Mg, Mg–6Zn, and Mg–5.4Zn–0.5Zr (ZK60) showed no adverse effect in the surrounding rat’s bladder tissue and no sign of toxicity towards the liver and the kidneys. Their degradation tests demonstrated that the pure Mg consistently corroded faster than the alloys in simulated body fluids and artificial urine and in the bladder of rats. However, those previous corrosion studies did not assess the formation of the corrosion layer. In reaction with the ionic content of urine, the surface of the ureteral stent can be easily calcified. Known as encrustation, this is one of the main problems currently faced with respect to ureteral stents [6]. Therefore, this work aims at revealing a detailed in vitro degradation behavior of Zn alloys under simulated urinary tract conditions by using an electrochemical method and advanced surface characterization techniques.

## 2. Material and Methods

### 2.1. Material and Specimen Preparation

A group of binary Zn alloys (Zn–0.5Mg, Zn–1Mg, and Zn–0.5Al) was selected based on their excellent mechanical properties as determined in our previous study [19]. Hot rolled pure Zn (99.995%) and pure Mg (99.94%) and commercially available extruded Mg–2Zn–1Mn alloy were used as comparative materials. The Zn alloys were melted at 500 °C in a cylindrical steel mold inside a resistance furnace (Nabertherm, Lilienthal, Germany) and cast as cylindrical billets of 80 mm length and 15 mm diameter. The billets were annealed at 350 °C for 48 h, water-quenched, and extruded to a final diameter of 8 mm as described in detail elsewhere [19]. Specimens for corrosion tests were cut from the extruded metals at a thickness of 4 mm using a slow speed diamond rotating blade (Isomet 1000 Precision Saw, Buehler, Uzwil, Switzerland). The specimens were then polished using SiC paper from #600, to #800, and to #1200 (Carbimet 2 Abrasive Paper, Buehler, Uzwil, Switzerland).

### 2.2. In Vitro Degradation Testing

Four methods of electrochemical corrosion tests were conducted: open circuit potential (OCP), electrochemical impedance spectroscopy (EIS), potentiodynamic polarization (PDP), and electrochemical noise (EN). The metal specimens were mounted as working electrodes in a waterproof chamber with an exposed area of 0.096 cm^2^ (0.35 cm of diameter), and all tests were done in triplicate for statistical analysis. The first three methods were performed one after another on all metal specimens using a three-electrode cell configuration. The metallic samples served as the working electrode, a graphite rod as the counter electrode, and a saturated calomel electrode (SCE) (E = +0.241 V saturated) as the reference electrode. The electrodes were connected to a VersaSTAT3 potentiostat and monitored using the VersaStudio software (Ametek Princeton Applied Research, Oak Ridge, TN, USA). The OCP experiments were conducted by monitoring the potential versus the SCE without applying any outside source for 3600 s, until equilibrium was reached at the corrosion potential E_corr_. Once the equilibrium was achieved, EIS was started at the amplitude of 10 mV RMS and a frequency scan from 100 kHz to 1 Hz for all specimens. The interpretation of EIS results was made with the help of ZSimpWin software (Ametek Princeton Applied Research, Oak Ridge, TN, USA). The PDP experiments were done following the EIS, using a scan rate of 0.6 V/h, from −0.25 to +0.6 V vs. OCP.

The EN experiments were conducted on pure Mg, pure Zn, and Zn–0.5Al specimens only. Two identical working electrodes (mounted in resin) and a saturated silver chloride electrode (E = +0.197 V saturated) reference electrode were used and connected to a PC4/750 galvanostat/potentiostat (Gamry Instruments, Warminster, PA, USA). Data processing was done using an electrochemical signal analyzer (ESA400, Gamry Instruments, Warminster, PA, USA). The EN analysis was performed independently from the other electrochemical tests, as different equipment was used. The EN experiments were conducted for 48 h and at a frequency of acquisition of 10 Hz. The two identical coupled WEs were connected to a zero resistance ammeter (ZRA) to maintain the bias potential at 0 V. Each set of EN analysis, containing 1024 data points, was recorded with a data sampling rate of 0.1 s.

The artificial urine solution was made from analytical grade chemicals (Sigma-Aldrich, St. Louis, MO, USA) following the composition presented in Table 1. The pH of the artificial urine solution was adjusted to 6.0 using a solution of 1 N NH_4_OH and monitored with a pH meter (Accumet pH meter 25, Fisher Scientific, Portsmouth NH, USA). To mimic the ureteral condition, the solution was kept at a normal human body temperature of 37 ± 1 °C using a water heater and a jacketed beaker. It was stirred with a magnetic stirrer at 80 rpm. The artificial urine was chosen over natural urine to allow more reproducible results. The natural variability of the chemical composition of urine is greatly inconsistent between individuals because of the different diet and physical activity level, and it also depends on the time of the day, with more concentrated urine in the morning [20].

### 2.3. Surface and Corrosion Layer Characterization

The surface morphology observation and chemical composition analysis of the specimens after PDP were done using a scanning electron microscope (SEM, Quanta 250 FEI, Hillsboro, OR, USA) coupled with an energy dispersive X-ray spectroscope (EDS, SwiftED 3000, Oxford Instruments, Concord, MA, USA). The specimens were rinsed with distilled water and dried in desiccator for one day before being subjected to SEM observation. An X-ray photoelectron spectroscope (XPS, PHI 5600-ci spectrometer, Physical Electronics, Chanhassen, MN, USA) was then used to further determine the nature of the corrosion products at the surface of the corroded samples. A neutralizer was used to negate the charging effect at the surface of the metals. An XPS spectra survey was recorded from 1400 eV and ending at 0 eV using a standard aluminum anode (1486.6 eV) at 300 W. High-resolution spectra were then collected using a standard magnesium anode (1253.6 eV) at 300 W aimed at the following elements: carbon (300–280 eV), oxygen (545–525 eV), zinc (1037–1017 eV), phosphorus (147–127 eV), magnesium (64–44 eV), and, finally, aluminum (88–68 eV). The C1s peak was chosen as a reference at 285.0 eV to negate the effects of charging. Both survey and high-resolution scans were done in triplicate for each metal.

## 3. Results and Discussion

**Figure 1:**
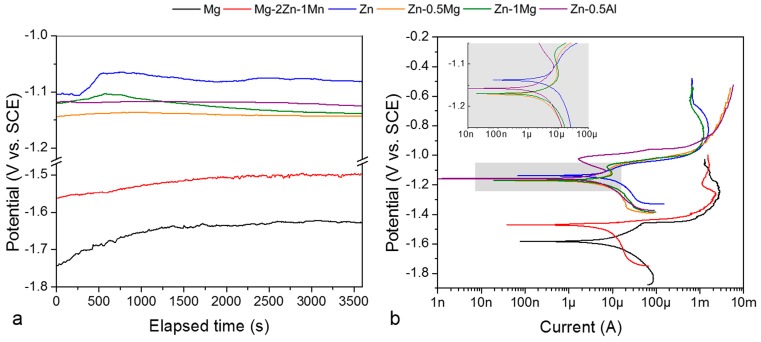
Typical open circuit potential (OCP) and potentiodynamic polarization (PDP) results: (**a**) potential vs. time, (**b**) potential vs. current.

Once immersed in the artificial urine solution, all metals tended to stabilize after 1 h (Figure 1a). Pure Mg and Mg–2Zn–1Mn showed a more negative open circuit potential (−1.76 and −1.52 V, respectively) compared to all Zn groups (between −1.15 and −1.05 V with pure Zn having the highest value). Under potentiodynamic polarization, the Zn group shows a more noble behavior compared to the Mg group (Figure 1b). Corrosion rates vary from 2.16 mm/year for pure Mg to 0.87 mm/year for Zn (Table 2). The Mg–2Zn–1Mn corrodes slightly slower than pure Mg at 1.90 mm/year due to alloying effects. The corrosion of magnesium is highly dependent on the impurities that can act as active cathodic sites to accelerate corrosion. Zn is nobler than Mg and may act as an impurity, but the addition of Mn increases the tolerance limit of impurity’s content by reducing the grain size of the alloys, thus lowering the corrosion rate of Mg–2Zn–1Mn compared to that of pure Mg [22,23,24]. In the Zn group, corrosion rate increases from pure Zn to Zn–0.5Al and Zn–Mg (Table 2). The addition of Mg into Zn produces an Mg_2_Zn_11_ phase that creates microgalvanic action with the Zn matrix, which in turn increases the corrosion rates of Zn–Mg alloys [25]. Meanwhile, the high solubility of Al in Zn produces a single phase microstructure in the Zn–0.5Al, so its corrosion rate only slightly increases [19].

**Figure 2:**
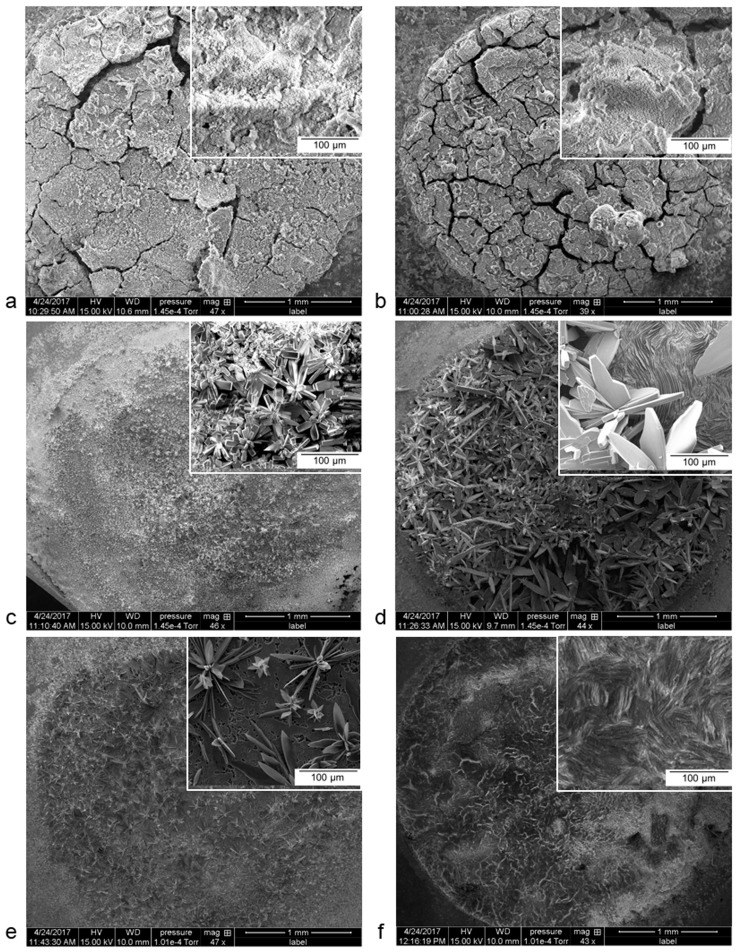
SEM images of the specimens’ surfaces after polarization: (**a**) pure Mg, (**b**) Mg–2Zn–1Mn, (**c**) pure Zn, (**d**) Zn–0.5Mg, (**e**) Zn–1Mg, and (**f**) Zn–0.5Al.

After the polarization test, the surface of Mg group specimens is covered by a fairly uniform but not seemingly compact corrosion layer as indicated by the presence of cracks (Figure 2a,b). A pure Zn surface presents a uniform and dense corrosion layer (Figure 2c), as it tends to passivate in a near neutral aqueous solution, such as artificial urine [26]. Both the investigated Zn–Mg alloys show a non-uniform corrosion layer with the appearance of a flower-like crystal on the surface. The flower size is larger in Zn–0.5Mg (Figure 2d) with a seemingly compact underlying layer compared to that of Zn–1Mg (Figure 2e), which could explain the slightly faster corrosion rate of the latter. The flower is not observed on the Zn–0.5Al surface, but a rather uniform, smooth, and dense corrosion layer (Figure 2f).

The distinct difference on the corrosion layer’s composition between the Mg group and the Zn group is the calcium content, which was only detected on that of the former group with a concentration range of 13–15 wt % (Table 3). The Mg group’s composition has similarity to that of Mg alloys reported in many non-urinary in vitro and in vivo studies, and it generally forms hydroxide, phosphate, and carbonate [23,27,28,29,30,31]. Apart from the lack of calcium, the EDS spectra of the Zn group corrosion layer showed a lower content of oxygen and phosphorous, but a higher carbon content as compared to the Mg group. A focused EDS analysis on the flower-like formation on the surface of pure Zn and Zn–Mg alloys (Figure 2c–e) reveals a high content of zinc and oxygen, which supposedly forms ZnO, as later confirmed by XPS analysis. On the very top layer, as measured by the XPS, both groups have a similar content of carbon and oxygen, but a higher phosphorous content for the Zn group. Apart from the aluminum content, the surface of the Zn–0.5Al alloy has the lowest content of those elements, as measured by both techniques. Previous non-urinary in vitro and in vivo corrosion studies of pure Zn generally confirmed the formation of zinc oxide, zinc carbonate, zinc phosphate, and CaP on the very top layer [14,15,19,32,33]. The presence of calcium on the corrosion layer may initiate calcification and thus encrustation, a main current problem of ureteral stents [6], leading to the formation of urinary stones. These stones are composed mainly of calcium oxalate (CaO_x_), struvite (NH_4_MgPO_4_·6H_2_O), calcium phosphate (CaP), and uric acid (C_5_H_4_N_4_O_3_) [34,35].

**Figure 3:**
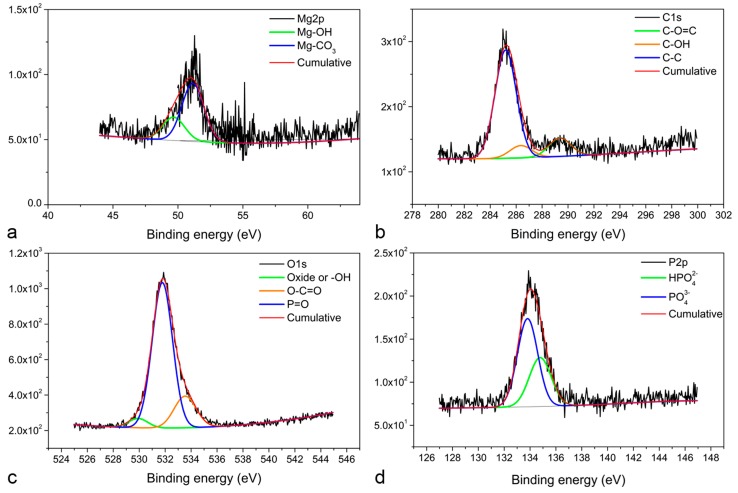
High resolution XPS spectra for pure Mg specimen: (**a**) Mg2p peaks, (**b**) C1s peaks, (**c**) O1s peaks, and (**d**) P2p peaks.

Further XPS analysis allowed for the observation of two Mg2p peaks at 51.15 and 49.66 eV on pure Mg and at 51.19 and 50.07 eV for Mg–2Zn–1Mn (Figure 3a), which correlate to MgCO_3_ and Mg(OH)_2_, respectively [36,37]. Three C1s peaks (Figure 3b) are also detected on these specimens at 285 eV for C–C, 286.6 eV for C–O(H) [38], and 289.7 eV for carbonate [37]. Three O1s peaks (Figure 3c) are present at 530.15 eV related to MgO or Mg(OH)_2_, 531.7 eV for the double binding of P=O [39], and at 533.3 eV related to O–C=O of CO_3_ species such as MgCO_3_ or CaCO_3_ [40]. Additionally, two P2p peaks were observed at 133.6 and 135 eV (Figure 3d), which correspond to MgHPO_4_ and Mg_3_(PO_4_)_2_, respectively [41].

**Figure 4:**
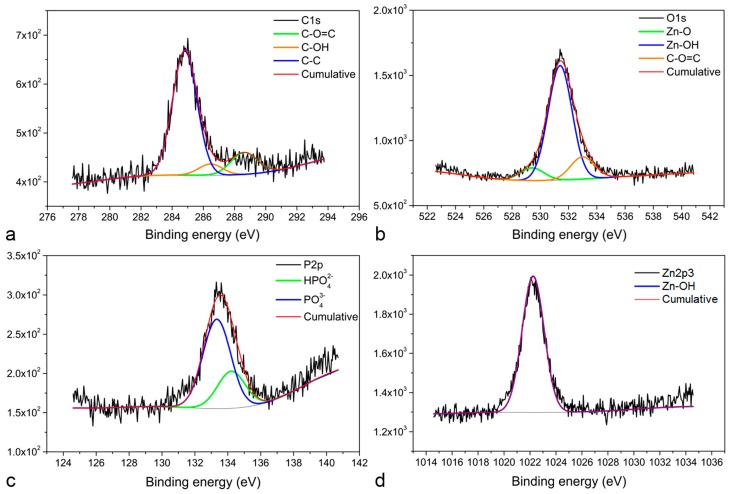
High resolution XPS spectra for Zn–0.5Al specimen: (**a**) C1s peaks, (**b**) O1s peaks, (**c**) P2p peaks, and (**d**) Zn2p3 peak.

Regarding the Zn–0.5Al alloy, the XPS analysis demonstrates the presence of three C1s peaks related to C–C at 285 eV, carbonate at 289.15 eV, and C–O(H) at 286.89 eV (Figure 4a). The first O1s peak was observed at 532 eV and corresponds to Zn(OH)_2_ [42], the second at 529.6 eV is related to ZnO [42], and the third at 533 eV fits to carbonate (Figure 4b). Two P2p peaks were observed at 133.47 and 134.31 eV (Figure 4c), characterized as ${\mathrm{PO}}_{4}^{3-}$ and ${\mathrm{HPO}}_{4}^{2-}$ [41], which confirms the presence of a phosphate compound in the corrosion layer. One peak was observed for Zn2p3 at 1022.8 eV (Figure 4d), which corresponds to Zn(OH)_2_ [42]. An additional peak of Al2p (not shown) was found at 74.76 eV for Al_2_O_3_ [43]. Overall, the XPS analysis confirms that no calcium-related compound was formed on the surface of Zn–0.5Al, which makes this alloy less susceptible to calcification compared with pure Mg and the Mg–2Zn–1Mn alloy.

**Figure 5:**
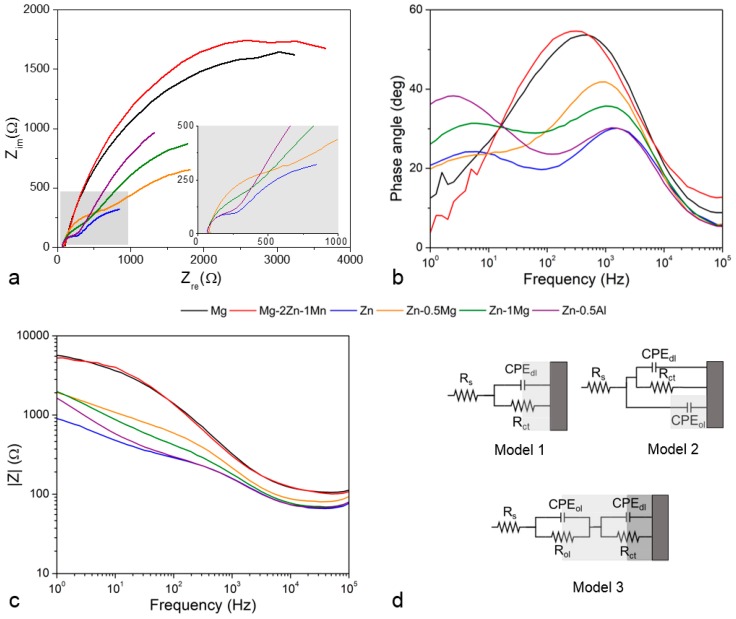
Typical electrochemical impedance spectroscopy (EIS) results: (**a**) Nyquist plots, (**b**) Bode phase angle plots, (**c**) Bode impedance plots, and (**d**) proposed equivalent electrical circuit models. Note that at very low frequency the Mg specimens show some negligible scatter.

The corrosion layers formed on the metals’ surface exhibit a different impedance behavior, as indicated by their Nyquist plots (Figure 5a). The Mg group’s plots form a semi-circle (one time constant), whilst those of the Zn group show two semi-circles (two time constant). Looking at the Bode plots, in the Mg group the phase angles increase from about 10° to 55° at a high frequency and then decrease to about 25° at a lower frequency (Figure 5b), indicating a capacitive loop [44]. Meanwhile, the plots for the Zn group show two peaks, one at a high frequency and another at a low frequency, except for the Zn–0.5Mg where the second peak is not that apparent. In the other Bode plots, all specimens show similar evolution from low impedance at high frequency to high impedance at low frequency, with the Mg group having the highest impedance (Figure 5c), indicating the corrosion layer exerts an electric effect between a resistor and a capacitor.

By applying a single-frequency voltage to the interface, EIS allows a measurement of the electric properties of the corrosion layer, including a phase shift, a real and imaginary part of the impedance and the amplitude of the resulting current at that particular frequency [45,46]. All these properties can be simplified as an electrical circuit with each electrical component representing one of the elements of the electrochemical cell. For the Mg group, their semi-circle plots indicate the formation of a typical uniform oxide layer (Figure 2a,b). Its characteristic can be fitted with a simple electrical circuit (Model 1 (Figure 5d)) composed of the R_s_ (solution’s resistance), R_ct_ (charge transfer resistance), and CPE_dl_ (constant phase element) of the double layer at the electrolyte–surface interface. The CPE represents a non-ideal electrical element in the circuit; in this case, it behaves as an imperfect capacitor and is very useful for assessing its dispersive effect at the surface of the electrode [47]. For a corroding metal, this effect is attributed to the microscopic increase in the surface roughness of the electrode [48]. When R_ct_ is the only resistance in the circuit, it can often be substituted with R_p_ (polarization resistance) and it is inversely proportional with the corrosion rate. For the Zn group, its oxide layer characteristic is fitted with the Model 2 circuit that includes a CPE_ol_ for the capacitive properties of the oxide layer, considering that Zn is less reactive than Mg and formed a rather non-uniform corrosion layer (Figure 2c–f). A more complex electrical circuit can be fitted to all specimens, representing a bi-layered surface with outer CPE_ol_ and R_ol_ (Model 3). The calculated value of each electrical component from the equivalent circuits is presented in Table 4.

The solution’s resistance, R_s_*,* is in the constant range of 5–10 Ω throughout all the experiments. As shown in the Model 1 and Model 3 circuits, the Mg group exhibits a higher R_ct_ compared to the Zn group, conversely to their CPE_dl_. The addition of Mn may play a role in improving the coating resistance of the Mg–2Zn–1Mn by increasing the passivity of the corrosion layer [28]. Both Zn–0.5Mg and Zn–1Mg exhibit similar electrical values, so in EIS analysis they are treated as one metal. The presence of a large value of CPE_ol_ (Model 2) indicates that a preferential corrosion occurred on the surface of the Zn–Mg alloys, leading to the creation of micropores on the corrosion layer (Figure 2e). The CPE_dl_ value of the Zn group is higher than those of the Mg group due to the higher capacitive properties of Zn, which produces fewer active ions than magnesium, and this could be the reason for the slower degradation rate of the former [49]. The value decreases from pure Zn to Zn–0.5Al and to Zn–Mg, meaning more charge should be accumulated at the electrode-electrolyte interface of pure Zn since no element acts as a captor of charged ions, i.e., Mg in Zn–Mg and Al in Zn–Al in the process of localize corrosion. In the Model 3 circuit, R_ct_ and CPE_dl_ are affected by the interaction of different layers, which in turn changes the behavior of both components. The R_ol_ value is much higher for the Mg group, which could be correlated to a thicker oxide layer (Figure 2a,b). The capacitive properties of the oxide layer are bound with CPE_ol_. In this bi-layer model, the oxide layer of pure Zn and Zn–0.5Al has dielectric properties, which could be influenced by a thick and dense corrosion layer governed by the diffusion phenomenon (Figure 2c). However, this may not be the case for Zn–Mg alloys as the layer becomes less uniform due to the localized corrosion (Figure 2d,e).

**Figure 6:**
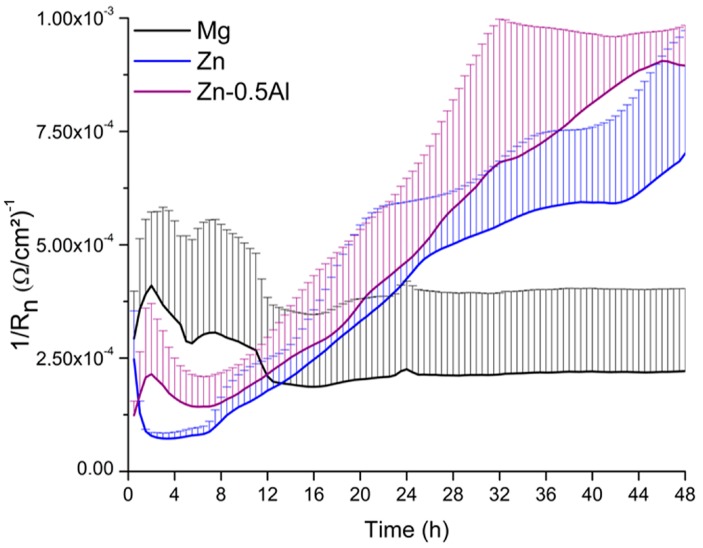
Electrochemical noise (EN)results presented as 1/R_n_ vs. time for pure Mg, pure Zn, and the Zn–0.5Al alloy over a period of 48 h.

The electrochemical noise data were analyzed in the time domain with the calculation of the noise resistance R_n_. It is well established that R_n_ is inversely proportional to the corrosion rate [50]. This helps to monitor the corrosion rates after immersion of the specimens in the urine solution. Pure Mg, pure Zn, and Zn–0.5Al were used as samples for the EN investigation. Up to 12 h of immersion, pure Mg exhibited the highest corrosion rate followed by Zn–0.5Al and pure Zn (Figure 6), a similar trend was obtained by the accelerated PDP test. Pure Mg experienced a stabilization after 12 h, so, for longer periods, pure Mg possesses the lowest corrosion rate than the other two metals.

**Figure 7:**
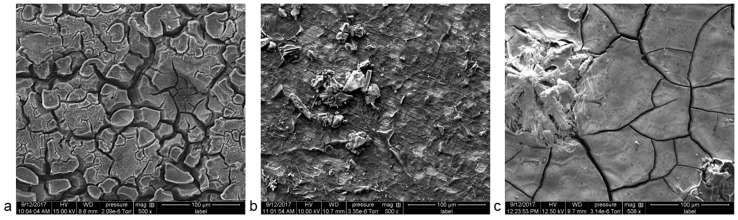
Surface morphology after 48 h of the EN test of (**a**) pure Mg, (**b**) pure Zn, and (**c**) Zn–0.5Al.

In artificial urine, pure Mg dissolves quickly. Due to the high affinity of Mg ions with the ions in the solution, an insoluble corrosion layer (i.e., Mg_3_(PO_4_)_3_) forms and protects the metals underneath from further corrosion [18]. This thick layer (Figure 7a) acts as a more effective barrier than Mg(OH)_2_ or Zn(OH)_2_ on pure Zn [17]. A rather smooth surface was observed on the latter (Figure 7b). The Zn–0.5Al is prone to microgalvanic corrosion due to the potential difference of the Zn and Al phases (Figure 7c).

## 4. Conclusions

In artificial urine, the studied pure Mg and its alloy show a corrosion behavior distinct from that of pure Zn and its alloys. Based on the accelerated potentiodynamic polarization test, the Mg group exhibits a higher corrosion rate than does the Zn group. The corrosion layer of the Zn group has a more capacitive behavior than that of the Mg group. A flower-like structure of Zn oxide forms on the polarized surface of the Zn group, except for Zn–0.5Al, while precipitation of Ca and Mg was observed in the Mg group only. In the long-term electrochemical noise test, pure Mg exhibits stabilization after 12 h and thus has an overall lower corrosion rate than does pure Zn and Zn–0.5Al. The continuous metal dissolution of Zn–0.5Al and the low affinity to ions in artificial urine prevents the formation of a thick corrosion layer. In view of ureteral stent applications, a continuous dissolution of metal in urine, such as the case of the Zn–0.5Al alloy, is ideal to prevent calcification (stent encrustation) and bacterial adhesion on the surface.

## Figures and Tables

**Table 1:** Artificial urine composition [21].

Chemical	NaCl	NaH_2_PO_4_	Na_3_C_6_H_5_O_7_	MgSO_4_	Na_2_SO_4_	KCl	CaCl_2_	Na_2_C_2_O_4_
Mass (g)	6.17	4.59	0.944	0.463	2.408	4.75	0.638	0.043

Brought to 1 L by adding distilled water.

**Table 2:** Corrosion parameters derived from PDP results.

Sample	OCP (V vs. SCE)	i_corr_ (µA/cm²)	Corrosion Rate (mm/year)
Mg	−1.58 ± 0.02	94 ± 37	2.16 ± 0.84
Mg–2Zn–1Mn	−1.48 ± 0.07	84 ± 17	1.90 ± 0.38
Zn	−1.11 ± 0.06	58 ± 6	0.87 ± 0.09
Zn–0.5Mg	−1.18 ± 0.01	92 ± 5	1.39 ± 0.07
Zn–1Mg	−1.17 ± 0.01	99 ± 5	1.50 ± 0.08
Zn–0.5Al	−1.15 ± 0.01	77 ± 2	1.14 ± 0.03

**Table 3:** Elemental composition of the corrosion layer detected by EDS and XPS.

Sample	Element						
**EDS (wt %)**	**C**	**O**	**Mg**	**P**	**Ca**	**Zn**	**Al**
Mg	4.9	50.6	11.9	19.7	13.0	-	-
Mg–2Zn–1Mn	2.3	47.2	13.3	22.1	15.3	-	-
Zn	16.4	17.5	-	8.8	-	57.3	-
Zn–0.5Mg	13.9	11.7	-	7.9	-	66.6	-
Zn–1Mg	22.3	14.2	-	5.6	-	57.9	-
Zn–0.5Al	9.2	6.7	-	4.0	-	65.5	14.6
**XPS (at %)**	**C1s**	**O1s**	**Mg2p**	**P2p**	**Ca2p**	**Zn3p2**	**Al2p**
Mg	31 ± 10	40 ± 8	15 ± 2	5 ± 4	9 ± 1	-	-
Mg–2Zn–1Mn	23 ± 3	46 ± 3	14 ± 4	9 ± 12	8 ± 3	-	-
Zn	25 ± 3	45 ± 2	-	18 ± 2	-	12 ± 2	-
Zn–0.5Mg	28 ± 5	42 ± 5	-	19 ± 1	-	11.1 ± 1	-
Zn–1Mg	31 ± 6	42 ± 2	-	17 ± 3	-	10 ± 2	-
Zn–0.5Al	26 ± 2	44 ± 2	-	9 ± 7	-	10 ± 2	5 ± 2

**Table 4:** EIS-derived parameters (R in Ω cm² and CPE in µF).

Specimen		Mg	Mg–2Zn–1Mn	Zn	Zn–Mg	Zn–0.5Al
Model 1 R(QR)	R_ct_	634 ± 141	1357 ± 321	-	-	-
	CPE_dl_	56 ± 8	277 ± 49	-	-	-
Model 2 R(Q(QR))	R_ct_	-	-	9 ± 4	5 ± 5	13 ± 2
	CPE_dl_	-	-	8480 ± 2770	1220 ± 1700	2740 ± 153
	CPE_ol_	-	-	23 ± 15	2030 ± 1520	29 ± 10
Model 3 R(QR)(QR)	R_ct_	45 ± 1	104 ± 47	20 ± 3	25 ± 4	495 ± 108
	R_ol_	716 ± 178	583 ± 47	95 ± 27	242 ± 51	14 ± 2
	CPE_dl_	85 ± 11	84 ± 11	2970 ± 1060	871 ± 119	74 ± 17
	CPE_ol_	63 ± 19	317 ± 101	77 ± 48	82 ± 49	1850 ± 170
